# Cross-organ MRI to assess the relationship between cardiac traits and cerebral white matter hyperintensity volumes

**DOI:** 10.1038/s43856-026-01604-8

**Published:** 2026-04-23

**Authors:** Adama Fatima Saccoh, Nikhil Paliwal, Sam Quill, Albert Henry, Marion van Vugt, Carole H. Sudre, A. Floriaan Schmidt

**Affiliations:** 1 Department of Population Health and Experimental Medicine, Institute of Cardiovascular Science, Faculty of Population Health, University College London, London, United Kingdom; 2 Garvan-Weizmann Centre for Cellular Genomics, Garvan Institute of Medical Research, Sydney, NSW Australia; 3 Faculty of Medicine and Health, University of New South Wales, Sydney, NSW Australia; 4 Department of Cardiology, Amsterdam Cardiovascular Sciences, Amsterdam University Medical Centre, University of Amsterdam, Amsterdam, The Netherlands; 5 Hawkes Institute, Department of Computer Science, University College London, London, United Kingdom; 6 School of Biomedical Engineering & Imaging Sciences, King’s College London, London, United Kingdom

**Keywords:** Magnetic resonance imaging, Epidemiology

## Abstract

**Background:**

Cardiac disease is linked to early cognitive decline and dementia, with shared risk factors such as hypertension, inactivity, and *APOE4* genotype. White matter hyperintensities (WMH) may mediate this association.

**Methods:**

We investigated associations between 29 cardiac MRI (CMR) measures and 5 regional WMH volumes independently of known CVD risk factors. We analysed 30,961 UK biobank participants without pre-existing cardiovascular disease (CVD) or known dementia. We empirically validated the CMR traits by associating these to incidence CVD using Cox’s regression models. Linear regression models were used to identify association between 6 regional WMH volumes and cognitive tests outcomes (executive function and processing speed), as well as with 29 CMR traits.

**Results:**

Twenty-one CMR traits associate with heart failure and atrial fibrillation, 10 with coronary heart disease, and 4 with abdominal aortic aneurysm. Frontal and parietal WMH volumes associate with executive function and processing speed. We identify distinct groups of CMR associations where right heart traits (excluding RVEF) primarily associate with parietal WMH volumes, contrasting with the more widespread associations of left cardiac and aortic traits. Higher values of aortic areas, LV mass and wall thickness associate with higher WMH volumes for all brain regions. Furthermore, increased WMH volumes associate with increased minimum and maximum ascending aortic areas. We additionally observe associations between increased biventricular ESV and increased WMH volumes, with increased EF associating with lower WMH volumes.

**Conclusions:**

We show that CMR derived measurements of function and structure associate with regional WMH volumes implicated in executive functioning and processing speed, independently of known cerebrovascular risk factors in people without established disease.

## Plain Language Summary

Heart and brain ill-health share common risk factors such as high blood pressure, diabetes, smoking, and obesity. People with heart disease tend to experience greater brain changes related to blood vessel dysfunction such as white matter hyperintensities (WMH) in later life. White matter intensities are white spots visible when the brain is imaged by Magnetic Resonance Imaging (MRI) and are indicative of damage to the part of the brain called white matter. These changes are related to poorer cognition and higher risk of stroke and dementia. We investigated how measures of heart function and structure relate to WMH volumes. We found that right heart measurements have a more specific relationship than left heart and aortic measurements which were widespread in comparison. Our findings provide confirmatory evidence about the relationship between cardiac and brain MRI findings in people without established disease or cerebrovascular risk factors.

## Abstract (web summary)

Saccoh et al. investigate the associations between 29 cardiac MRI traits and regional brain white matter hyperintensity volumes (WMH) in disease free individuals. They show right heart traits (excluding RVEF) mainly associate with parietal WMH volumes in contrast to left cardiac and aortic traits that have widespread regional WMH associations.

## Introduction

Cardiovascular disease (CVD) and dementia are the leading causes of morbidity and mortality in the world^1^. Coronary heart disease (CHD) and cerebrovascular disease share common risk factors including high blood pressure, diabetes, smoking, and obesity^2^. Furthermore, individuals with established cardiac diseases such as CHD, atrial fibrillation (AF), and heart failure (HF) are at risk of early cognitive decline, which may progress towards dementia, for example due to cardiometabolic stroke, adverse vascular function, or cerebral atherosclerosis^2,3^.

With an aging population, that increasingly survives an initial cardiac event, a substantial number of individuals are at risk of developing early cognitive decline and dementia. The association between hypertension and poorer brain and cognitive health is widely appreciated^4^. Moreover, adherence to the American heart association (AHA) Life’s Simple 8, which represent the ideal cardiovascular health factors, is associated with lower white matter hyperintensity (WMH) volumes and longer late-in-life cardiometabolic disease-free life expectancy^5^. Previous studies on individuals with early-onset CVD(≤60 years) have identified associations between abnormal left cardiac structures and function, including left ventricle (LV) ejection fraction (EF), LV mass and left atrial volume (LAV), and factors like hypertension duration, increased WMH volumes, and poorer midlife cognitive function^6–8^.

These shared associations between cardiac abnormalities, as well as common risk factors for cardiac and cerebrovascular diseases, raise questions of whether change in cardiac function and structure directly impact brain health, or merely reflect common risk factors. For instance, irregular blood flow may contribute to ischemia in deep matter regions of the brain contributing to regions of WMH.

Furthermore, it remains unclear whether poorer cardiac function and/or structure are associated with brain health in individuals without established CVD or if this association is primarily driven by the presence of CVD itself. Similarly, the specific links between cardiac magnetic resonance (CMR) findings and WMH remain uncertain, particularly in relation to distinct cardiac or vascular diseases. For instance, associations between EF and HF, or between CMR-derived aortic measurements and abdominal aortic aneurysms (AAA) could provide insights into distinct pathogenetic mechanisms leading to WMH occurrence.

In this manuscript, we aim to determine the extent to which 29 CMR traits from the aorta and left and right heart chambers associate with brain magnetic resonance imaging (MRI) based measurements of five regional and total WMH volumes in individuals free of CVD and dementia at baseline. We first determine which CMR traits associate with the incidence of AAA, CHD, AF, and HF, as well as identify WMH volumes associating with cognitive function. Among the CMR traits linked to the incidence of cardiac disease, we examined their association with WMH volumes across multiple brain regions, especially focussing on frontal and parietal lobes. Finally, we explore difference in these association across participant subgroups based on age (less than 65 years compared to 65 years and older), sex, and *APOE4* carriership (heterozygous or homozygous).

We identify distinct groups of CMR associations where right heart traits (excluding RVEF) primarily associate with parietal WMH volumes, contrasting with the more widespread associations of left cardiac and aortic traits. These findings support a relationship between cardiac function and structure, and regional WMH volumes independently of known cerebrovascular risk factors in people without established disease.

## Methods

### Study sample

We leveraged data from the population-based UK Biobank (UKB) cohort^9^, and identified 30,961 individuals who participated in the cross-organ imaging sub-study. Common clinical and demographic covariates were extracted for each participant: age (years), sex, body mass index (BMI, kg/m^2^), height (cm), weight (kg), systolic blood pressure (SBP, mmHG) diastolic blood pressure (DBP, mmHG), low density lipoprotein cholesterol (LDL-C, mmol/L), total cholesterol, glycated haemoglobin (HbA1c, mmol/mol). Family (mother, father, siblings) history of Alzheimer’s, hypertension, stroke, and heart disease was categorised as binary variables. Socioeconomic factors of household income, Townsend Multiple deprivation index was obtained alongside educational status, which was coded to their highest level of education (See below).

Body surface area (BSA) was calculated for each participant using the Dubois formula^10^. Based on the available whole exome sequencing (WGS) we additionally identified participants who were heterozygous (*N* = 7835, 25%) or homozygous (*N* = 667,2%) for *APOE4* genotype (Supplementary Data 1, 2)

### Educational status

Educational status was defined using UK Biobank (UKB) field 10035. Participants were grouped as described below, where participants with “none of the above” or “no” answers were removed.

1. Completed college or university or having other professional qualifications, Advanced(A)levels or equivalent: A levels/Advanced subsidiary (AS) levels and equivalent includes the Higher School Certificate.
2. O levels or General certificate of secondary education (GCSE): O levels/GCSEs and equivalent include the School Certificate.
3. Certificate of secondary education (CSE) or equivalent

completed National Vocational Qualification (NVQ).– practical work-based awards given in England, Wales and Northern Ireland Qualifications.

### Identifying people without established disease

To prevent effects by pre-exiting conditions, we removed participants with a diagnosis of CVD or dementia prior to, or 30 days after, the imaging appointment, specifically we removed: 1597 individuals with CHD, 198 with HF, 715 with AF, 85 with AAA, and 69 individuals with any dementia. All cause dementia was extracted based on UKB field ID 42019. Given the small number of non-European participants (*n* = 1030) these were excluded (Supplementary Fig 1). In sensitivity analysis, we further removed participants with a diagnosis 365 days after imaging appointment (Supplementary Fig 2).

### Cognitive tests

At their imaging visit participants underwent a range of cognitive tests that can be mapped to performance in specific cognitive domains^11,12^ (Supplementary Table 1). The tests selected represent cognitive domains of executive function (alphanumeric trail making test and tower rearranging test) and processing speed (reaction time and symbol digit substitution test). Participants with no recorded time in the alphanumeric trail making test (0 s, *n* = 708) or beyond the test limit (5 min, *n* = 10) were excluded from the analysis.

### Incident cardiovascular events

Incident events of CHD, HF, AF, and AAA were recorded until October 2020, based on UK-wide linkage to hospital data provided by National Health Service Digital in England, Information and Statistics Division in Scotland, and Secure Anonymised Information Linkage in Wales. Date of event was defined as the date of first diagnosis. Full disease coding is listed (Supplementary Data 3).

### Ethics approval

Northwest Multi-Center Research Ethics Committee ethically approved the UK Biobank Study. Generic approval for the UK Biobank Study was granted by National Health Service (NHS) National Research Ethics Service (Ref. 11/NW/0382). This work was approved by the UK Biobank Ethnics Advisory Committee under application Number 12113(https://www.ukbiobank.ac.uk/about-us/how-we-work/ethics). No additional IRB approval was sought or required. All participants consent to the work done in accordance with UK Biobank agreement at enrolment.

### Derivation of cardiac and brain MRI traits

CMR images from the UKB were acquired using 1.5 Tesla. The MRI study protocol has been described in detail by Petersen et al.^9^. For CMR images, we processed the LV short-axis, long-axis, and aortic images. Each of these images was segmented using a pre-trained neural network to identify the aorta and the four cardiac chambers: left atrium (LA), right atrium (RA), LV and right ventricle (RV). Detailed methodology and validation of the segmentation model can be found in Bai et al.^13^. Based on the segmentation, we quantified a total of 29 cardiac traits, spanning the aorta (6 traits), atria (4/4 LA/RV), and| (11/8 LV/RV) ventricles, with details provided (Supplementary Table 2).

Brain MRI images from the UK Biobank were acquired using 3 T scanners. WMH Volumes were derived as described in Sudre et al.^14^. Using T1-weighted images, the cortical grey matter was automatically parcellated using Geodesic Information Flows, a label fusion algorithm, that also provides total intracranial mask and subject-specific tissue atlases^15^. Using these maps to provide anatomical information, BaMoS, a Gaussian mixture model jointly describing healthy tissue and unexpected signal was run on the co-registered T1-weighted and FLAIR images. After convergence, connected components of candidate lesion voxels were classified as either lesion or artefact resulting in a lesion probability map. A subject-specific regional map was created to localise WMH dividing the white matter (WM) and deep grey matter (DGM) volume into lobes and layers^16^. These regions were defined using as distance the solution for the Laplace equation between the ventricular surface and the cortical sheet. This was used to assign each voxel of the WM area not belonging to BG and Thalami to its closest cortical lobe defined as aggregated cortical region from the parcellation. Specifically, WMH volumes were derived over five brain regions: Frontal[F], Parietal[P], Temporal[T], Occipital[O], Basal Ganglia + Thalami as well as total WMH volume.

### Statistical analysis

Spearman’s estimator was employed to determine the correlation between CMR traits (Supplementary Data 4), among the six WMH volumes (Supplementary Data 5), as well as to establish the correlation between non-MRI covariates and CMR traits or WMH volumes (Supplementary Figs. 4, 5).

We identified the CMR traits associating with incidence cardiac disease as empirical validation. Specifically, we employed Cox’s proportional hazards regression to identify associations with the time from imaging to AAA, CHD, AF, or HF, or end of follow-up. These models were adjusted for cardiovascular risk factors (SBP, DBP, HDL-C, LDL-C, total cholesterol, smoking status, BSA, socioeconomic factors (household income, educational status, Townsend score and family history of disease (Alzheimer’s, Parkinson’s, high blood pressure, stroke, heart disease).

We then determined the association between total and brain region-specific WMH and executive function and processing speed. For this we regressed each test (alphanumeric, tower rearranging, reacting time and symbol digit substitution) results on each WMH volumes using a linear regression model adjusted for total intracranial volume (TIV), age at imaging visit, difference between age at initial visit and imaging visit, sex, SBP, DBP, HbA1c, smoking status, BSA, household income, educational status, Townsend score and family history of disease (Alzheimer’s disease, Parkinson’s disease, hypertension, stroke, or heart disease).

We set out to determine the association between WMH volumes adjusted for total intracranial volume (TIV), and CMR traits, regressing each WMH measurement in turn on an individual CMR trait. Estimates were adjusted for the same covariates used in the regression analysis with cognitive function. Subgroup analysis and interaction tests^17^ were conducted for: homozygous or heterozygous for *APOE4*, sex and age groups 65 years and above, and 45–64 years, with 45 years representing the youngest participants enrolled in the sub-study.

The primary analysis, the association between CMR traits and cerebral WMH volumes, was corrected for multiple testing applying a Bonferroni correction based on the number of principle components (PCs) needed to explain 90% of the CMR variability^18^; (Supplementary Fig. 6). Specifically, we found that 10 PCs were needed resulting in a multiple testing corrected *p*-value of 0.005. Prior to conducting the regression analyses the CMR and WMH measurements were mean centred and divided by the trait standard deviation (SD, Supplementary Data 6, 7). The linear regression results are presented in their original unit of measurements(i.e. for the cognitive score analysis), or as percentage mean difference per SD (i.e., applying the exponential function on the linear regression point estimates and confidence interval limits), where an estimate of 1 represents an absence of effect. Associations with onset of disease are represented as hazard ratios (HR). All results are presented with 95% confidence intervals (95% CI).

## Results

Data were available from 30,961 participants, including 16,537 female participants (53.4%), with a mean age of 65 (SD: 7.58) as shown in Table 1. The median total WMH volume is 1731 mm^3^(quartile (Q), Q1 1039; Q3 3510) for female participants and 1792 mm^3^ (1056; 3775) for male participants. The median WMH volumes are highest in the frontal lobe (862 mm^3^; 432; 1955) with smaller volumes in the subsequent lobes. The parietal lobe (170 mm^3^ 70; 507) the temporal (222 mm^3^ 132; 406), occipital (235 mm^3^ 125; 431) and basal ganglia and thalami regions (171 mm^3^ 105; 275). Regional WMH volumes excluding basal ganglia and thalami are highly correlated (coefficients greater than 0.8 for parietal and 0.9 for frontal lobe) with total WMH (Supplementary Data 5).

**Table 1:** Participant characteristics of 30,961 individuals contributing to the UK Biobank cardiac and brain MRI measurements

Characteristics	
Sex, Female *N* (%)	16,537 (53.4)
Age (years), mean ±SD	65.05 (7.58)
BMI (kg/m^2^), mean ±SD	26.36 (4.01)
HDL-C (mmol/L), mean ±SD	1.49 (0.38)
LDL-C (mmol/L), mean ±SD	3.59 (0.82)
Total cholesterol (mmol/L)), mean ±SD	5.73 (1.06)
Hba1c (mmol/mol), mean ±SD	34.79 (4.86)
SBP (mmHg), mean ±SD	135.99 (18.44)
DBP (mmHg), mean ±SD	81.13 (10.41)
Smoking status *N* (%)	
Ever smoked	10,104 (32.63)
Never smoked	18,948 (59.74)
Townsend score, median (Q1, Q3)	−2.66 (−3.91, −0.61)
Educational attainment *N* (%)	
University, College, Professional Qualifications	19,109 (61.62)
A level	2552 (8.27)
O levels or GCSE	4664 (15.03)
CSE	1226 (3.98)
NVQ	1451 (4.71)
Household income (£) *N* (%)	
<18,000	3120 (10.94)
18,000–30,999	5909 (20.72)
31,000–51,999	8631 (30.27)
52,0000–100,000	8504 (29.84)
>100,000	2344 (8.22)

The presented numbers reflect counts (percentages), mean (standard deviation), median (quartile 1: Q1, quartile 3: Q3). Household income represents income groups based on UK Biobank field 738.

*BMI* body mass index, *HDL-C* high density lipoprotein cholesterol, *LDL-C* low density lipoprotein cholesterol, HbA1c Haemoglobin A1c, *SBP* systolic blood pressure, *DBP* diastolic blood pressure.

**Fig. 1:**
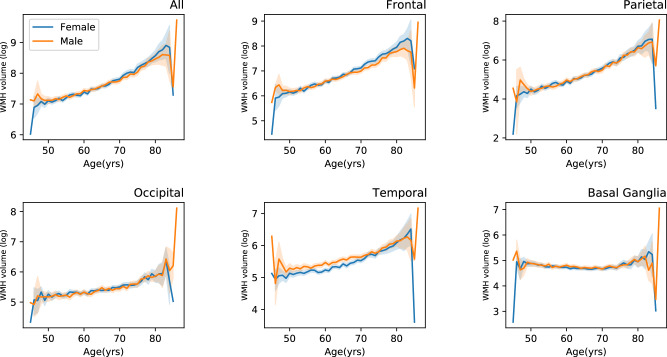
The mean white matter hyperintensity (WMH) volume by age and sex. The WMH volume is stratified by brain region as well as total. Analyses are based on 30,961 UK Biobank participants of European ethnicity, without a history of CVD or any dementia. Blue line represents female and orange line represents male. The shaded regions indicate the mean ±standard deviation. yrs years.

As shown in Fig. 1, WMH volume increased with age, but did not differ between men and women. WMH was additionally moderately influenced by blood pressure and Hba1c (Supplementary Fig. 7).

### The association between white matter hyperintensity volumes and cognitive function

Performance in cognitive test scores decreases with age (Supplementary Fig 3). For a subset of WMH volumes we determined their associations with cognitive test scores, shown in Table 2. The alphanumeric trail-making test and tower rearranging test were used to evaluate executive function. An increase in standard deviation of WMH volume associate with increased time for completing the alphanumeric test, specifically for total brain WMH: 0.77 s (95% CI 0.45; 1.10), frontal:0.74 s (95% CI 0.42; 1.08) and parietal volumes 0.62 s (95% CI 0.30; 0.95). No significant associations are found for the tower rearranging test (Supplementary Data 8). Symbol digit substitution and reaction time tests were used to evaluate processing speed. An increase in standard deviation of WMH volume shows an increase in reaction time of 2.68 milliseconds[ms] (1.40;3.97), for total brain WMH, 3.01 ms (1.74;4.31) frontal and 1.86 ms (0.58;3.13) parietal WMH volumes. Higher WMH volumes associate with a reduction in total correct symbol digit substitution results for total: −0.16 (−0.22; −0.10) and frontal: −0.16 (−0.20; −0.10) WMH volumes.

**Table 2:** The association between WMH volumes and cognitive test scores

Cognitive test (*N*)	Brain region	WMH (mm^3^) Mean (std)	Mean difference per standard deviation increase in WMH volume (95% CI)	*P*-value
Executive function				
Alphanumeric trail making (22,377)	Total (All regions)	3448 (4840)	0.78 (0.45;1.10)	3.12 ×10⁻⁶
	Frontal Lobe	1833 (2734)	0.75 (0.42;1.08)	9.07 ×10⁻⁶
	Parietal Lobe	650 (1468)	0.62 (0.30;0.95)	1.57 ×10⁻⁴
	Occipital Lobe	352 (399)	0.18 (−0.13;0.49)	2.46 ×10⁻¹
	Temporal Lobe	381 (525)	0.43 (0.12;0.75)	6.93 ×10^−^^3^
	Basal Ganglia & Thalami	230 (227)	0.22 (−0.09;0.52)	1.62 ×10^−1^
Processing speed				
Reaction time (29,035)	Total (All regions)	3344 (4699)	2.68 (1.40;3.97)	4.20 ×10^−5^
	Frontal Lobe	1761 (2648)	3.01 (1.74;4.31)	5.43 ×10^−6^
	Parietal Lobe	627 (1423)	1.86 (0.58;3.13)	4.21 ×10^−3^
	Occipital Lobe	348 (394)	1.12 (−0.08;2.32	6.86 ×10^−2^
	Temporal Lobe	379 (518)	0.822 (−0.414;2.05)	1.92 ×10^−1^
	Basal Ganglia & Thalami	228 (227)	0.56 (−0.63;1.75)	3.60 ×10^−1^
Symbol digit substitution (22,896)	Total (All regions)	3840 (4899)	−0.16 (−0.22; −0.10)	1.26 ×10^−6^
	Frontal Lobe	1850 (2744)	−0.16 (−0.2;−0.10)	4.55 ×10^−7^
	Parietal Lobe	660 (1484)	−0.13 (−0.20;−0.07)	4.53 ×10^−5^
	Occipital Lobe	354 (403)	−0.03 (−0.90; 0.027)	3.16 ×10^−2^
	Temporal Lobe	383 (527)	−0.05 (−0.12;0.00)	5.83 ×10^−2^
	Basal Ganglia & Thalami	230 (238)	0.02 (−0.04;0.08)	3.06 ×10^−2^

Estimates were based on an analysis of a subset from 30,961 UK biobank participants with cognitive tests, leveraging linear regression analysis regressing cognitive test scores on WMH volumes adjusted for adjusts for total intracranial volume(TIV), age, difference between age at initial visit and imaging visit, sex, systolic and diastolic blood pressure, glycated haemoglobin (Hba1c), smoking status(ever/never smoked), BSA, household income, educational status, Townsend score and family history of disease (Alzheimer’s, Parkinson’s, high blood pressure, stroke, heart disease) as binary variables). The mean difference represents the change in 1) alphanumeric test score (seconds) 2) processing speed (milliseconds) 3) Symbol digit substitution number of completed tests in a minute(60 s) by one standard deviation (std) increase in WMH volume (mm^3^).

*95% CI* 95% confidence intervals.

### Associations of CMR traits and the incidence of cardiovascular disease

**Fig. 2:**
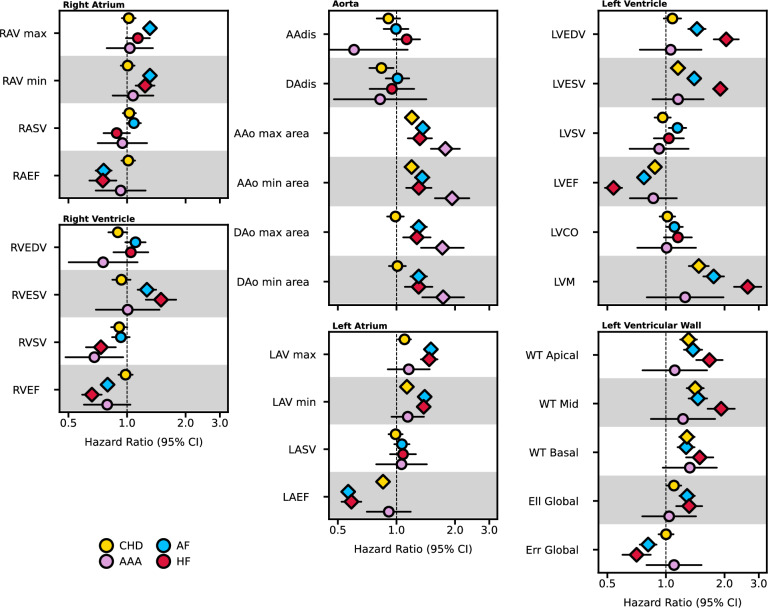
The cardiac MRI association with incidence cardiovascular disease. Analysis are based on 30,961 UK Biobank participants of European ethnicity, without a history of CVD or any dementia. For all CMR measurement, we determined their association with the onset of CHD, AAA, AF, HF using Cox’s proportional hazards regression. The measure of centre represents the hazard rations exponentiated coefficient and error bars the upper and lower 95% confidence intervals. The Cox models were adjusted for differences in cardiovascular risk factors (systolic and diastolic blood pressure, low density lipoprotein cholesterol, high density lipoprotein cholesterol, total cholesterol, smoking status (ever smoked/ never smoked), socioeconomic factors (household income, educational status, Townsend score and family history of disease (high blood pressure, stroke, heart disease). Results were tested against a Bonferroni correct *p*-value of 0.005, where diamonds represent *p*-values that are over the threshold and circles below threshold. Diseases are represented by colours; yellow-CHD, blue -AF, red-HF and pink AAA. CHD coronary heart disease, AAA Abdominal aortic aneurysm, AF atrial fibrillation, HF heart failure. Full list of cardiac trait abbreviations on the Y-axis can be found in Supplementary Table 2.

The Cox’s proportional hazards regression between CMR traits and onset of CHD, AAA, AF, or HF show that the majority of LV, LA, and aorta CMR traits associate with the onset of HF and AF, for example lower LVEF 0.54 (95% CI 0.49; 0.59), RVSV 0.73 (95% CI 0.62; 0.88) associate with increased HF while lower RAEF, 0.76 (95% CI 0.69; 0.83) and RVESV 1.27 (95% CI; 1.13; 1.41) associate with an increased risk of AF. The onset of CHD is predicted by LV mass 1.48 (95% CI 1.31; 1.66) apical wall thickness 1.31 (95% CI 1.18; 1.44), minimum ascending aortic areas 1.20 (95% CI 1.10; 1.30) and LAEF 0.85 (95% CI 0.79; 0.92). The maximum ascending 1.78 (95% CI 1.50; 2.12) and descending aorta 1.73 (95% CI 1.35; 2.22) associate with onset of AAA, (See Fig. 2 and Supplementary Data 9).

### Associations between CMR traits and white matter hyperintensity volumes

As described in the methods, we apply a 30- and 365-days windows of cardiac disease removal to which there were minimal difference and high correlation (0.99) between coefficients (Supplementary Fig. 2 and Supplementary Data 11). Therefore, we report the results of the 30-day removal window.

As shown in Supplementary Fig. 7, covariate adjustments has limited influence on the CMR association with WMH volumes, which did not differ by brain region. As such, we focus on the results for model 3 – adjusted for age, sex, cardiovascular risk factors, socioeconomic risk factors as well as family histories of neurodegenerative diseases (Methods).

**Fig. 3:**
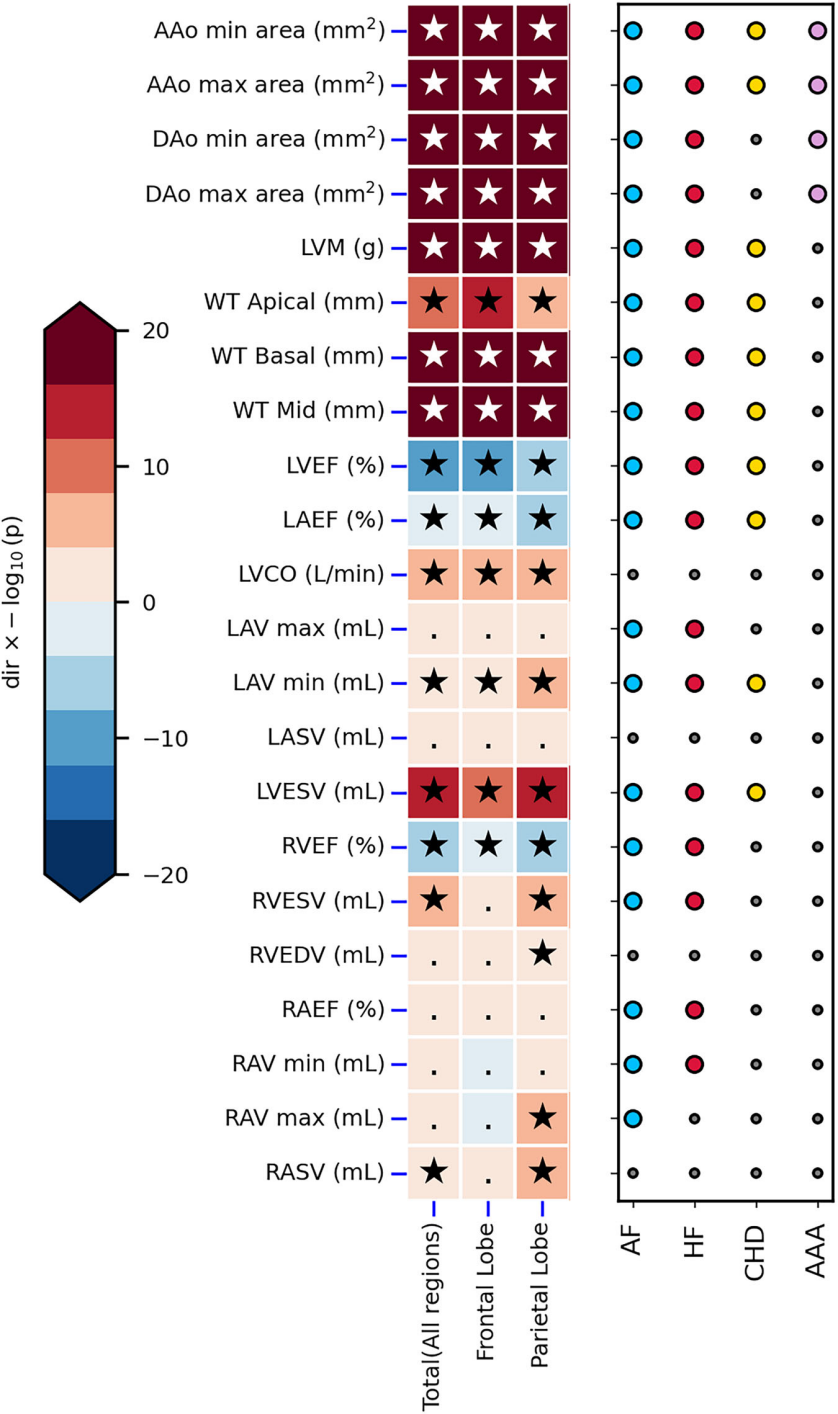
Associations between cardiac MRI traits and regional WMH volumes ranked by the cardiac MRI associations with incident cardiac disease. Analyses are based on 30,961 UK Biobank participants of European ethnicity, without a history of CVD or any dementia. The presented results were based on a linear regression model where in turn the total and regional WMH volumes were regressed on a CMR trait, accounting for the following covariates: age, sex, SBP, DBP, LDL-C, total cholesterol, smoking statues, household income, educational status, Townsend score, family history of disease (high blood pressure, stroke, heart disease) (see methods). Both the heatmap and the incidence matrix depicting the cardiac MRI associations with incident cardiovascular disease were ranked by the number of diseases the cardiac MRI trait was associated with. Results are presented as effect direction multiplied by the -log10(*p*-value), truncated to a maximum of 20, with blue representing a negative direction and red representing positive direction. Both cardiac trait values and WMH volumes were standardised, and results represent directionally based on 1 standard deviation change in measurement. Results with a *p*-value smaller than 0.005 is indicated by a star symbol, with results above 0.005 indicated by a dot. Cardiac traits are represented on the y-axis, with full list of abbreviations available in Supplementary Table 2 Regional and Total WMH volumes are displayed on the x-axis. CHD coronary heart disease, AAA Abdominal aortic aneurysm, AF atrial fibrillation, HF heart failure. Full list of cardiac trait abbreviations on the Y-axis can be found in Supplementary Table 2.

We observe that in individuals without established CVD, CMR traits associating with multiple cardiac outcomes also strongly associated with WMH volumes across all three brain regions linked to worse executive function, (Fig. 3). We identify distinct groups of CMR associations where right heart traits (excluding RVEF) primarily associated with parietal WMH volumes, contrasting with the more widespread associations of left cardiac and aortic traits.

For example, higher minimum and maximum ascending aortic area (associated with AAA) showed associations with larger total brain WMH volume: Larger aortic areas are associated with an additional 9% total WMH for minimum and (95% CI 1.08; 1.11) and maximum (95% CI 1.07; 1.10) ascending aortic areas, respectively. Similar WMH volumes increasing associations are observed for the descending aortic area. LVM, basal WT, mid WT, apical WT (associated with incident AF, HF). For example, for total brain WMH volume: LVM shows a 15% increase (95% CI 1.13; 1.17), basal WT 9% (95% CI 1.08; 1.11), mid WT 13% (95% CI 1.11; 1.14), and apical WT 5% increase (95% CI 1.04; 1.07). Increased biventricular EF (associated with AF, HF and CHD) associate with smaller WMH volumes, for example 3 percent decrease (95% CI 0.96; 0.98) for LVEF, and 2 percent (95% CI 0.96; 0.99) for RVEF and parietal WMH volumes. Similar biventricular associations with WMH volumes are observed for ESV (associated with the incidence of HF): 5% increase (95% CI 1.04; 1.07) for LVESV, and 3% (95% CI 1.02; 1.05) for RVESV and parietal WMH volume. In general, we observe consistency between CMR association with WMH volumes across different brain regions, typically also including those not necessarily associated with executive function (Fig. 3 and Supplementary Data 10).

We observe significant differences with modest effect sizes for sex, where CMR association with WMH are more pronounced in women. We found that CMR associations with WMH volumes was stronger in people younger than 65 years of age. Differences by *APOE4* carriership are less pronounced, with little difference in associations between (heterozygous or homozygous) *APOE4* carriers and non-carriers; (Supplementary Results, Supplementary Figs. 8–10, Supplementary Data 12–15).

## Discussion

In the current study of individuals without established cardiac disease and dementia, we found regional differences in CMR-derived parameters associations with brain WMH volumes. Regional WMH volume analysis shows frontal and parietal WMH volumes associated with executive function and processing speeds. This CMR-WMH volume association persisted after accounting for known cardiac and neurological risk factors such as blood pressure, cholesterol, and family history of disease. We observed a consistent and strong association with effect sizes exceeding 5% per standard deviation increase, between greater aortic areas, increased LV mass and wall thickness measurements, and larger WMH volumes indicating changes in aortic areas and LV mass may lead to decreased cerebral perfusion. Additionally, we observed associations with effect sizes between 3 and 5% that increased EF associated with lower WMH volumes, while increased biventricular ESV associates with higher WMH volumes. In contrast to left cardiac and aortic traits that have widespread regional WMH associations, our findings show that right heart traits (excluding RVEF) mainly associate with parietal WMH volumes with effect sizes of 2–3%.

Across multiple cognitive tests focusing on executive function and processing speed –alphanumeric trail making test, reaction time and symbol digit substitution, our study showed greater total, frontal and parietal WMH volumes associated with reduced test performance. This association persisted after accounting for known risk factors such as age, blood pressure, socioeconomic factors, and family history of disease. Our results align with the current literature linking notably frontal WMH volumes to reduced executive function using multiple cognitive tests^19–21^. We now show that CMR indices of structure and function, from left and right heart as well as aortic areas, are strongly associated with these regional WMH volumes. As executive functioning is mainly linked to the frontal lobe^19^, we additionally show the results with tests of processing speed showing associations with frontal and parietal lobes. Our results align with evidence that regional CSVD burden in the temporal, parietal, and occipital lobes to be associated with reductions in processing speeds and parietal-occipital burden to be associated with a reduction in verbal memory^20,22^.

Vascular cognitive impairment because of small vessel disease may begin with structural or functional changes of the heart. The brain utilises ~20% of output from the heart, therefore, changes in cardiac function are known to influence brain function^23^. Previous studies have mainly linked a small subset of left-ventricle cardiac indices routinely used in clinical practice (e.g. LVEF, LVM)^24–26^ and linked these changes in WMH volume without providing direct evidence for potential links with WMH volumes and cognition. Left ventricular geometry has previously been associated to brain architecture and cognition^27^. We extend this by identifying seven LV indices (LVEF, LVM, LVCO, LVESV, and LV wall thickness) associated with WMH volumes in regions implicated in executive function and processing speed.

This study further identified key CMR-derived aortic traits that are associated with specific WMH regions linked to executive functioning and processing speed. These findings suggest that aortic changes—possibly reflecting reduced vascular elasticity—may contribute to greater vascular damage. Diminished elasticity impairs arterial accommodation to changes in pulsatile pressure, which prompt compensatory responses from smaller arterioles impacting WMH volumes^28^.

The right ventricle has been understudied in heart-brain research as most studies focus on mechanisms arising from the left heart. Our study uniquely identifies RVEF, RAV, and RVESV as important RV CMR parameters that associate with parietal WMH burden independent of systemic blood pressure. Increased RAV could indicate early signals of poor atrial emptying, which is relevant given the established link between atrial dysfunction, atrial fibrillation and increased WMH volumes^29,30^. Higher RVESV could also point towards poor emptying of the ventricle and reduced RV systolic function. In addition to our work, Navarro et al. report ‘normal’ RV internal diameter to be associated with increased WMH volumes using a cohort of 132 individuals^31^. Mean right arterial pressure in a cohort with chronic valvular disease also shows associations with increased WMH volumes^32^. Together, these associations indicate early monitoring of right heart function could provide further understanding into the mechanisms of the RV and WMH volumes. Our findings on the specific localisation of these associations to parietal WMH volumes highlights the importance of stratified analysis that many studies lack. These findings suggest further analysis, which could include functional studies and clinical studies focusing on lobar distributions of WMH to better understand these associations.

This study provides detailed analysis on 29 cardiac traits and regional brain WMH volumes with measurements in the same individuals free of cardiovascular disease and dementia at the time of MRI. Our study provides a unique insight into the lobe-specific relationship of WMH and cardiac traits describing different contributions of left, right and aortic traits to overall WMH distribution independent of known risk factors. Our results support the presence of a subclinical relationship between cardiac function and WMH volumes, as well as with executive functioning, supporting the importance of cardiac health for healthy brain functioning. These associations were stronger in midlife (45–65 years) compared to later in life (above 65 years), suggesting midlife as a critical age for to improve or maintain heart as well as brain health. While these observations about the importance of midlife health for prolonged healthy living have been made before for lifestyle-related factors, we now expand these observations to midlife CMR measurements of cardiac function and structure. Our findings suggest patients identified with subclinical measures of cardiac function such as lower LVEF that puts them in a borderline range, may be important to monitor in terms of cognitive function and possible later in life adverse brain changes. The localisation of right CMR traits to parietal lobe WMH volumes provides an area of focus for further study to understand the implications on cognition due to the limited amount of research in this area. Our findings are relevant to ongoing considerations about integration of cardiac and cerebral clinical referrals.

The possibility of a vascular explanation for the heart-brain relationship where vascular changes lead to the development of small vessel disease provides some insight into the possible physiological mechanisms involved where specific left traits and aortic traits in our study have a strong overall association with WMH distribution. The relationship between WMH volumes and cognition is well reported to have negative associations but WMH presence is not the only contributor to a decline in cognition. Other brain pathologies can also contribute to reduced cognition independent of vascular risk. In addition, location of WMH may also be indirectly related to a reduction in lobe-specific cognitive abilities as location according to depth (periventricular, mid, and deep) of WMH load can also determine the effect on cognition^33^.

There are some limitations in this study. First, our findings may not be readily extrapolated to people of non-white ethnicities as they were excluded in our analysis. Future studies should focus on non-Europeans to validate these findings. Second, we report a disease-free study sample based on a 30-day cut off window, free of outliers, which may not account for cases where a clear diagnosis had not been made. Third, our cognitive analysis focused on a subset with cognitive data, which reduced sample size and may not be representative for the entire UKB cohort – although we show very small differences in WMH volumes between the subset and main data sample. We focus on executive function and processing speeds as the primary measure of cognition, given that the study population exhibited the greatest WMH burden in the frontal lobe—a region strongly associated with executive function and attention. Importantly, cognitive associations with increased WMH volumes have relatively small effect sizes in clinical terms. This reflects our disease-free subsample, hence these small differences mark meaningful associations as they are still found. Additional cognitive tests and comparisons with diseased population could provide further validation on established relationships between WMH volumes and cognition. Importantly, given the cross-sectional nature of our study, observation of associations should not be interpreted causally. Longitudinal studies may shed more light in the underpinning mechanistic pathways linking heart and brain and could validate the vascular explanation for a relationship.

## Conclusions

This study utilises an extensive collection of 29 cardiac MRI indices in combination with cognitively relevant regional WMH volume measures and cognitive tests. We demonstrate that in people without pre-existing cardiovascular disease, and independently of known CVD risk factors, there are widespread and regional associations between changes in cardiac and aortic function and structure with WMH volumes.

## Supplementary information

Transparent Peer Review file

Supplemental Information

Description of Additional Supplementary files

Supplementary data

## Data Availability

Access to the UK Biobank participant data used is governed by an application process managed internally on review. UK Biobank data are available for use by eligible researchers from academic, charity, government and commercial organisations – across the world – for health-related research that is in the public interest- https://www.ukbiobank.ac.uk/use-our-data/apply-for-access/. Aggregated results in the figures and tables within the manuscript, including numerical source data for main text and supplementary information are provided in Supplementary Data 1.
