# Supplementary material for: Cross-organ MRI to assess the relationship between cardiac traits and cerebral white matter hyperintensity volumes
Source: Commun Med (Lond). 2026 Apr 23;6:359. doi: 10.1038/s43856-026-01604-8 (PMC13309535; doi:10.1038/s43856-026-01604-8)
Supplement: Supplementary file 2 — Supplemental Information [file 43856_2026_1604_MOESM2_ESM.pdf]

Supplementary

Cross-organ MRI to assess the relation between cardiac measurements and cerebral white matter hyperintensity volumes

Saccoh et al.

Supplementary Results

Interaction test to identify potential difference in the associations between cardiac traits and WMH volumes

Interaction tests were used to identify possible differences in the CMR association with WMH values by age, sex, and *APOE4* status (Supplementary Figure 8), Supplementary Data 12-15)

APOE4 carriership

The subgroup analyses by *APOE4* homozygosity or heterozygosity provided limited evidence to support a difference in CMR-WMH volume association (Supplementary Data 12-13). People with a *APOE4* homozygous mutation had a slightly more pronounced decreasing association of aorta distensibility measurements (AAdis, DAdis) and WMH volumes of the occipital lobes.. The heterozygous analysis additionally identified a small difference between longitudinal strain (Ell global) association with WMH volume of the frontal lobe.

Age differences

Generally we observed a larger association between CMR indices and WMH volumes in people aged between 45-64; (Supplementary Figure 9, Supplementary data 14). These differences were minor with a minimum/maximum change of 5%. LVEDV, LVSV, LVCO and Err global showed a stronger association with frontal WMH volumes in the 45-64 age group. The parietal lobe associations were also stronger in 45-64 age group for distensibility, aortic areas and mid and basal wall thickness.

Sex differences

We observed a minimum change of 3% and maximum change of 7% between sex groups where CMR-WHM volume associations were mostly greater in females (Supplementary

Figure 10, Supplementary Data 15). Frontal lobe associations for LVESV, LVM, aortic areas and mid wall thickness was greater in females. Parietal lobe associations showed stronger associations in LVEDV, LVSV, LVM, RAV max, RASV and wall thickness for males. LVEF associations were attenuated for females.

Supplementary Table 1: UK Biobank Cognitive Tests

UKB Field ID	Test	Evaluation	Cognitive domain
23324	Symbol digit substitution test	Number of correct tests completed in a minute	Processing speed
20023	Reaction time	Mean time to identify correct matches – measured in milliseconds	Processing speed
21004	Tower rearranging test	Total number of puzzles current.	Executive function
6350	Alphanumeric test	Time taken to complete originally measured in deciseconds – converted to seconds	Executive function

A description of UK Biobank cognitive tests, how they are evaluated and the cognitive domain they represent administered in the current study

Supplementary Table 2: Abbreviations for all the cardiac traits with a description of their characteristics

Cardiac Trait	Characteristic
Right Ventricular Stroke Volume (RVSV)	Function
Right Ventricular End-Diastolic Volume (RVEDV)	Structure
Right Ventricular End-Systolic Volume (RVESV)	Structure
Right Ventricular Ejection Fraction (RVEF)	Function
Right Atrial Stroke Volume (RASV)	Structure
Right Atrial Volume at Maximum (RAV max)	Structure
Left Atrial Stroke Volume (LASV)	Function
Left Atrial Ejection Fraction (LAEF)	Function
Left Atrial Volume at Minimum (LAV min)	Structure
Left Atrial Volume at Maximum (LAV max)	Structure
Left Ventricular Stroke Volume (LVSV)	Function
Left Ventricular Ejection Fraction (LVEF)	Function
Left Ventricular Cardiac Output (LVCO)	Function
Left Ventricular End-Diastolic Volume (LVEDV)	Structure
Left Ventricular Mass (LVM)	Structure
Ascending Aorta Minimum Cross-Sectional Area (AAo min area)	Structure
Ascending Aorta Maximum Cross-Sectional Area (AAo max area)	Structure
Descending Aorta Minimum Cross-Sectional Area (DAo min area)	Structure

Descending Aorta Maximum Cross-Sectional Area (DAo max area)	Structure
Ascending Aorta Distensibility (AAdis)	Function
Descending Aorta Distensibility (DAdis)	Function
Basal Wall Thickness (WT Basal)	Structure
Mid Wall Thickness (WT Mid)	Structure
Apical Wall Thickness (WT Apical)	Structure

Supplementary Figure 1 The study flow chart of UK biobank participants with cardiac MRI and brain MRI measurements

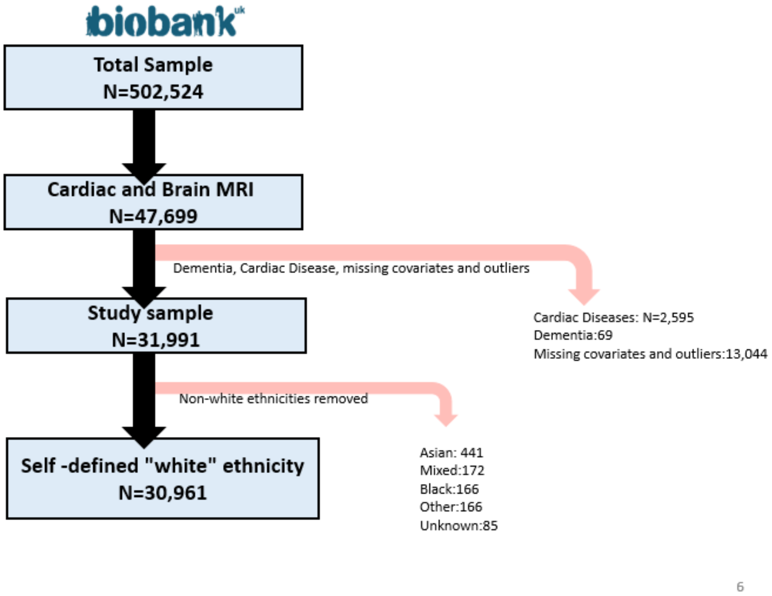

In the UK Biobank 47,699 participants had both cardiac and brain MRI images. We excluded participants based on baseline diagnosis of dementia, coronary heart disease, abdominal aortic aneurysm, atrial fibrillation, or heart failure (see Supplementary Table 3)for diagnostic codes). We removed outliers for cardiac disease where measurements were not biologically representative. We additionally removed people with missing covariate measurements, which

included : age (years), sex, body mass index (BMI, kg/m^2), height (cm), weight (kg), systolic blood pressure (SBP, mmHG) and diastolic blood pressure (DBP, mmHG), low density lipoprotein cholesterol (LDL-C, mmol/L), total cholesterol, glycated haemoglobin (HbA1c,mmol/mol), family (mother, father, siblings) history of Alzheimer's, hypertension, stroke and heart disease as binary variables. Socioeconomic factors of household income, Townsend score and educational status, where participants were coded to their highest level of education Due to the relatively small number of participants of non-white ethnicity, these were excluded.

Supplementary Figure 2 30 / 365 day window removal of cardiac diseases comparison of beta coefficients of CMR and WMH volumes

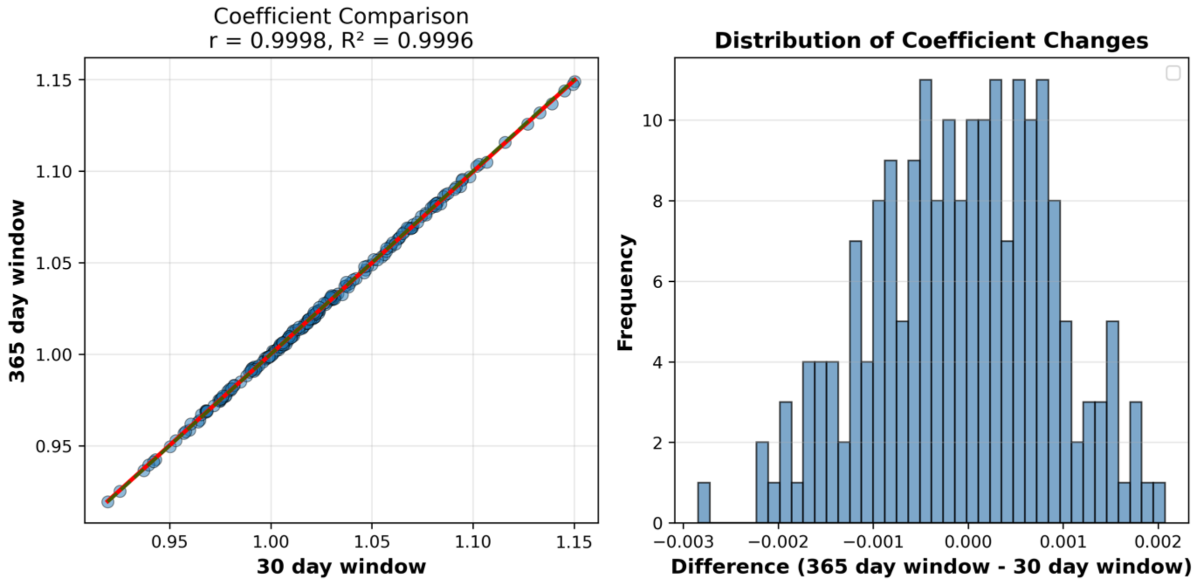

We have carried out additional analysis applying a 365 day window which subsequently removes 1,745 individuals with CHD, 230 with HF, 832 with AF, 103 with AAA. This had minimal effect on our originally reported results,

Supplementary Figure 3 Cognitive test performance by age

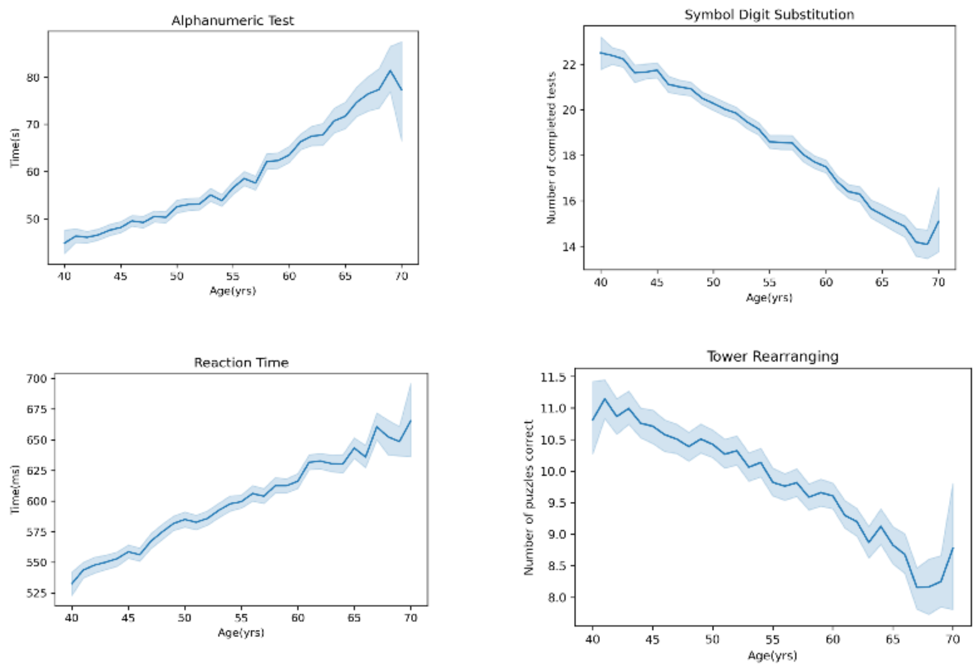

Time is measured in seconds and age in years. Participants with test times of 0 seconds (n=743) and longer than 5 minutes (n=11) in the alphanumeric test judged to be unrealistic, potentially representing coding mistakes, and were excluded

Supplementary Figure 4 Spearman's correlation between cardiac traits and covariates

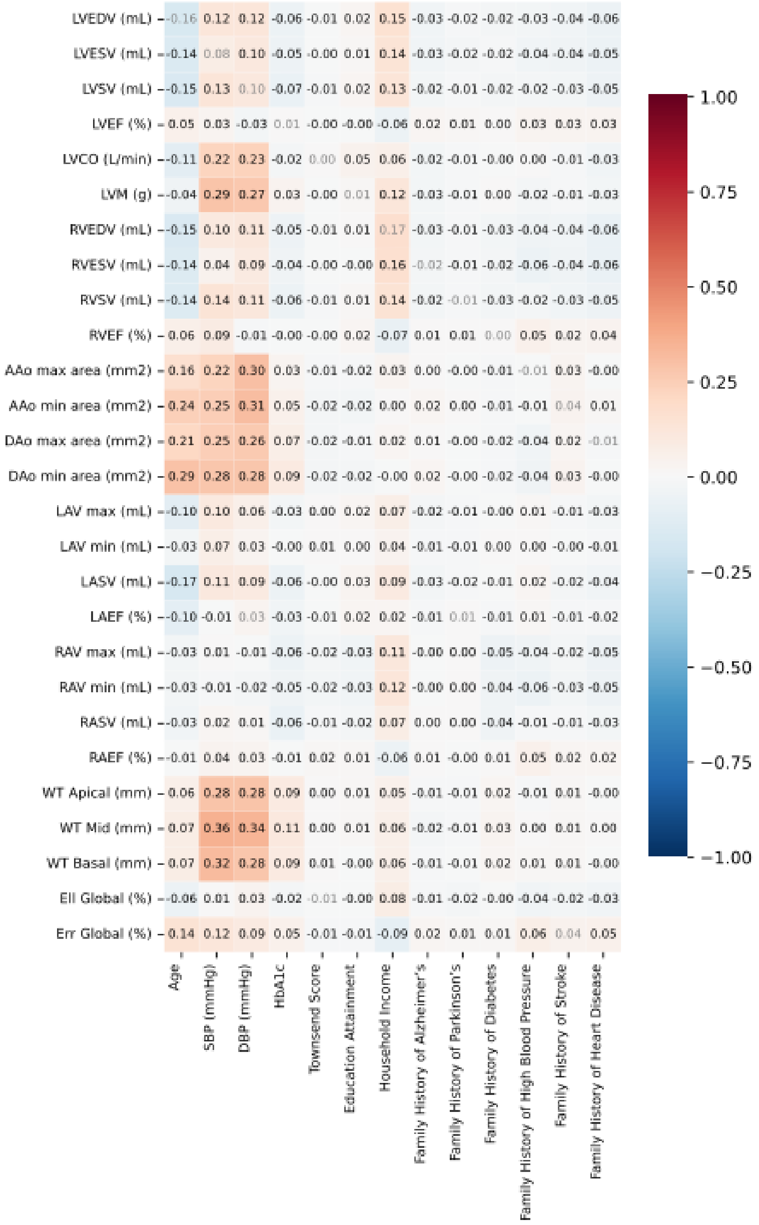

Spearman's estimator coefficients for cardiac traits and covariates. Associations of ($p<0.05$) are presented in black and ($p>0.05$) presented in grey For full abbreviations of cardiac traits (Supplementary Table 2)

Supplementary Figure 5 Correlation Matrix between White Matter Hyperintensity Volumes and covariates

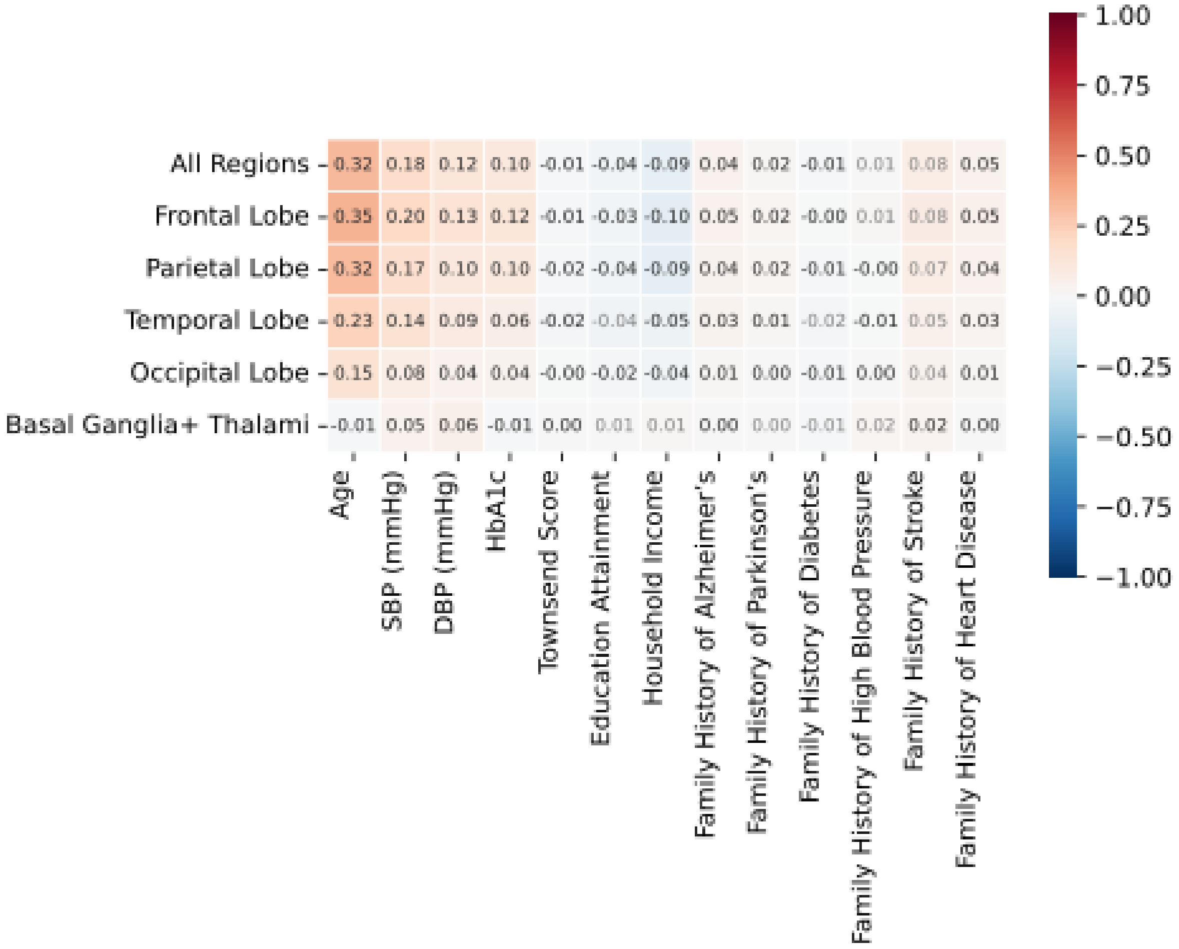

Spearman's estimator coefficients for brain traits and covariates. Associations of (p<0.05) are presented in black and (p>0.05) presented in grey. For full abbreviations of cardiac traits (Supplementary Table 2)

Supplementary Figure 6 The cumulative explained variance of cardiac MRI based principle components

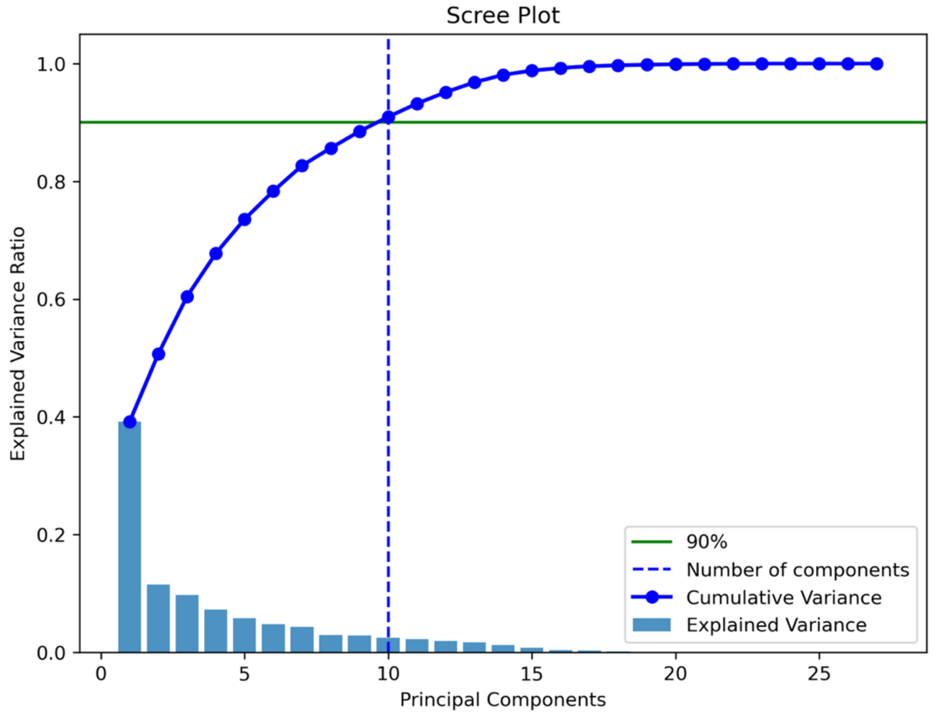

A scree plot showing Principle component (PCs) analysis was used to determine the number of components necessary to explain at least 90% of the CMR variability, representing the multiplicity burden

Supplementary Figure 7 The associations between cardiac traits and total WMH volumes with increasing covariate adjustment.

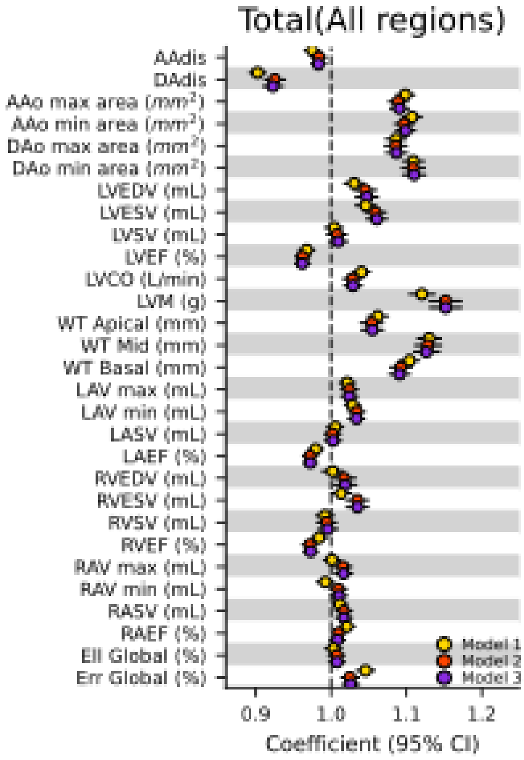

Total WMH volumes was regressed on each individual CMR measurements and adjusted for : model 1) age, difference between age at initial visit and imaging visit, sex ,model 2) additionally adjusted for SBP, DBP, Hba1c, smoking status, BSA, and model 3) including further adjustments for household income, educational status, Townsend score and family history of disease (Alzheimer's disease, Parkinson's disease, high blood pressure, stroke, heart disease).Abbreviations for cardiac traits (Supplementary S4)

Supplementary Figure 8: Interaction test p-values for potential difference in the associations between cardiac traits and WMH volumes

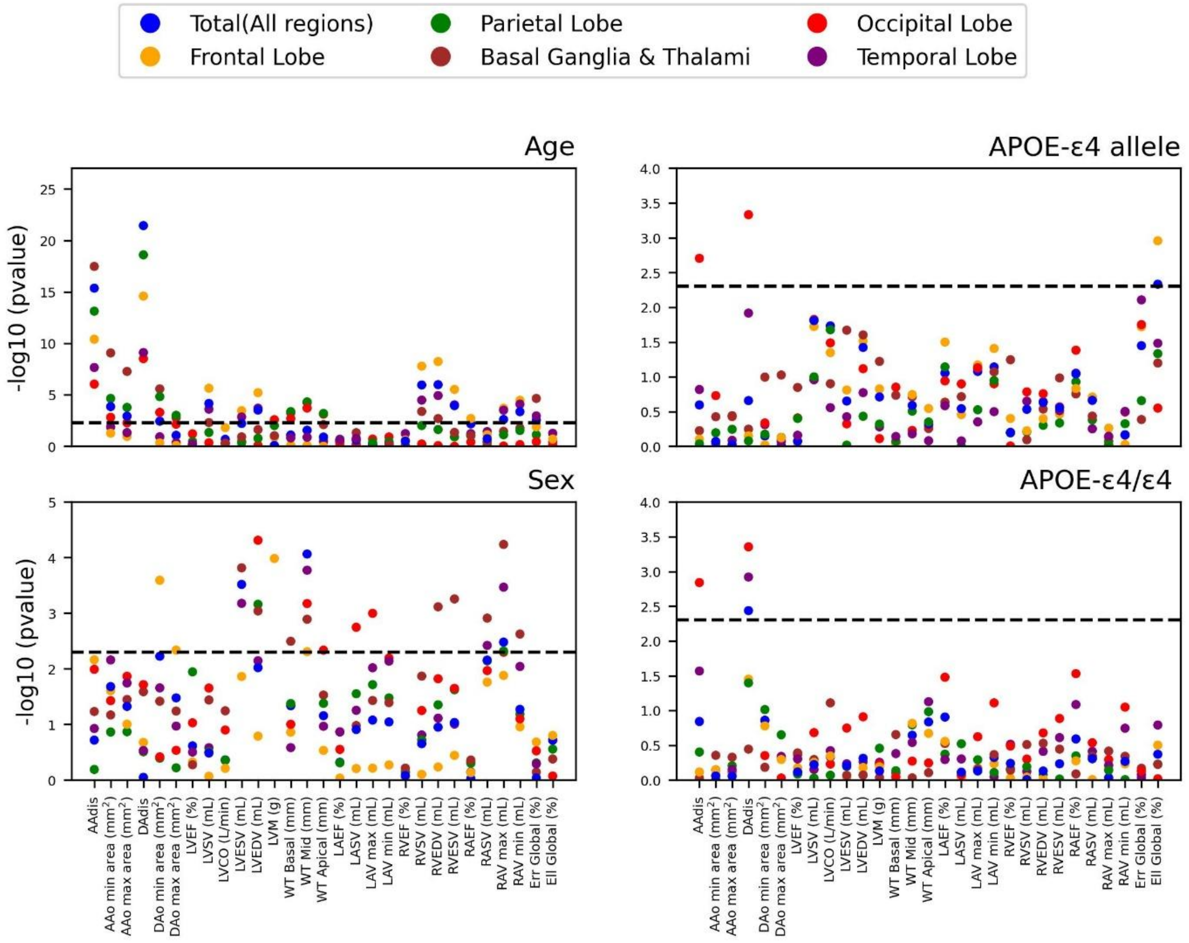

The interaction p-value evaluates potential difference in CMR-WMH volumes by key risk factors of cognitive decline: age (split on 65 years), sex (male and female) and *APOE4* carriership (homozygous, heterozygous). The dashed black line signifies the Bonferroni corrected p-value (0.005).

Supplementary Figure 9: Difference in associations of WMH and cardiac traits for age groups split on 65 years(45-64years and 65-85years)

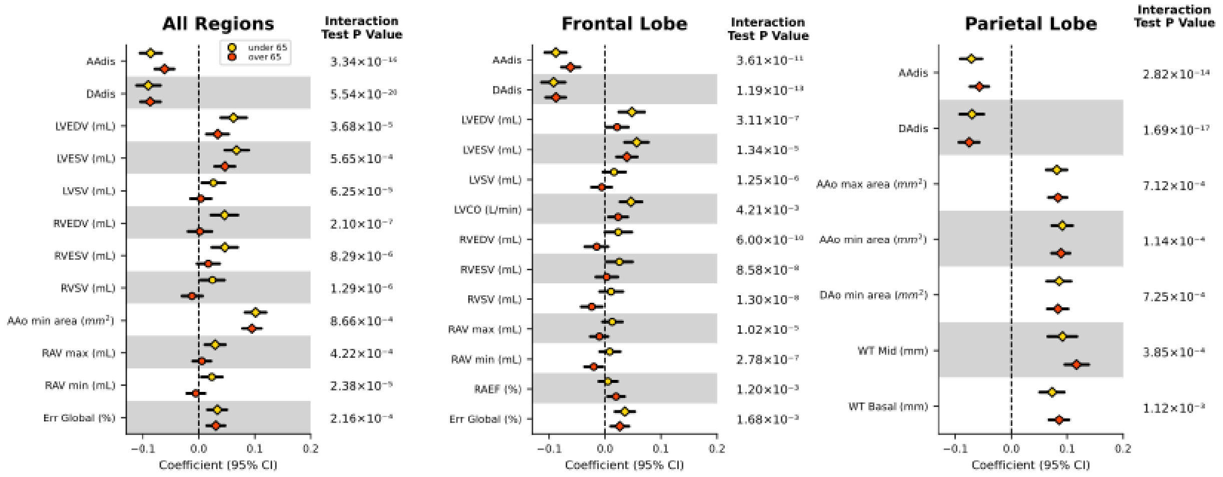

The association between cardiac traits and WMH volumes between age groups split on 65 years, 45-64years and 65-85years. The associations were adjusted for age, difference between age at initial visit and imaging visit, sex, SBP, DBP, Hba1c, smoking status, BSA, and socioeconomic factors including household income, educational status, Townsend score and family history of disease (Alzheimer's disease, Parkinson's disease, high blood pressure, stroke, heart disease). Significant interactions are represented as diamonds and coefficients less than are represented as circles. P-values presented in the table represents the ANCOVA interaction test significance between groups. Significance is based on Bonferroni (0.005) correction of P-values. Abbreviations for cardiac traits (Supplementary Table S4).

Supplementary Figure 10: Linear Regression coefficients of sex groups with significant interaction test values.

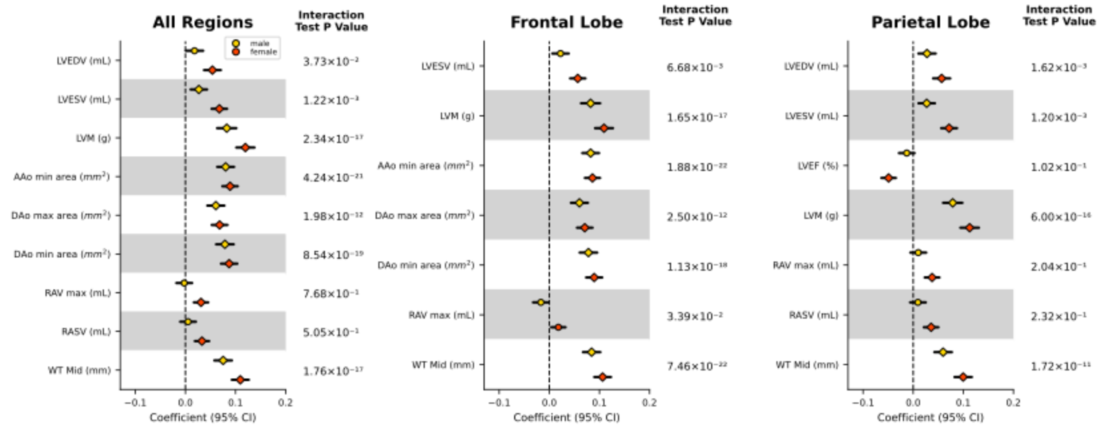

The association between cardiac traits and WMH volumes between sex groups , male and female. Male= yellow and female=orange. The associations were adjusted for age, difference between age at initial visit and imaging visit, sex, SBP, DBP, Hba1c, smoking status, BSA, and socioeconomic factors including household income, educational status, Townsend score and family history of disease (Alzheimer's disease, Parkinson's disease, high blood pressure, stroke, heart disease). Significant interactions are represented as diamonds and coefficients less than are represented as circles. P-values presented in the table represents the ANCOVA interaction test significance between groups. Significance is based on Bonferroni (0.005) correction of P-values. Abbreviations for cardiac traits(Supplementary Table S4).
