# Supplementary material for: Cross-organ MRI to assess the relationship between cardiac traits and cerebral white matter hyperintensity volumes
Source: Commun Med (Lond). 2026 Apr 23;6:359. doi: 10.1038/s43856-026-01604-8 (PMC13309535; doi:10.1038/s43856-026-01604-8)
Supplement: Supplementary file 1 — Transparent Peer Review file [file 43856_2026_1604_MOESM1_ESM.pdf]

nature portfolio

Peer Review File

Cross-organ MRI to assess the relationship between cardiac traits and cerebral white matter hyperintensity volumes

Corresponding Author: Ms Adama Saccoh

This file contains all reviewer reports in order by version, followed by all author rebuttals in order by version.

Version 0:

Reviewer comments:

Reviewer #1

(Remarks to the Author)
This is a well designed, large population based, cross sectional, associational study of cardiac and brain MRI features and cognition. The UK population is well defined and documented, the MRI and image analysis methodology sound and appropriate. The statistical analysis is likewise appropriate for contemporary 'big data' applications. The results show expected statistical relationships between cardia MRi variables, age, and CVD; and brain MRI variables (White Matter Hyperintensities) and cognition. In addition, statistically significant associations were shown between cardiac and brain MRI variables. While the latter results are relatively novel and worth disseminating, their practical significance and inferred clinical utility are quite unclear and weaknesses of the paper.

First, the effect size, while statistically significant, is relatively small with its practical significance only speculative. This is a not uncommon problem of large population studies with many variables in which some statistically significant results are almost inevitably found, depending on the setting of significance thresholds and multi-test adjustments. Perhaps to a fault, this reviewer views such results skeptically, wanting additional rationale for any clinical inference. This is particularly important for cross sectional studies where any inference of causality is very limited, as acknowledged by these authors.

A step in this supportive direction is often mechanistic, i.e. pathophysiological. The results fit some biological explanatory model. I am not aware of well documented anatomic, physiological, or biochemical mechanisms linking a particular right heard function to white matter integrity in the parietal lobe, though the authors do refer to a vague hypoperfusion hypothesis.

While acknowledging causal weaknesses of cross sectional studies, these, as well as many authors, proceed to causal arguments, to the extent of treatment suggestions or implications. How might one treat a specific right heart dysfunction that would directly and independently ameliorate parietal white matter deterioration, and cognition. It is particularly difficult to appreciate the clinical significance of such a weak mechanism and its treatment in light of the far larger and general effect of hypertension which still requires attention.

In short, this methodologically sound paper provides some new, primarily exploratory, information worthy of dissemination, but with limited mechanistic rationale and illdefined clinical significance.

Reviewer #2

(Remarks to the Author)
The work provides an important assessment for an expanded group of CMR markers against WMH with a division into different brain regions. The most notable finding seems to be a difference with right heart CMR measures localizing to the parietal lobe.

The main area I think could be improved upon is in the statistical analysis. There is a gargantuan amount of stats being run looking at associations but I only see a mention of a division by 10 for correction. Given the equally gargantuan sample size

of the study I think that clinically meaningful associations should withstand a more robust multiple comparisons correction taking into account 29 predictors and 5 outcomes. ALternatively, once a positive association is shown for total wmh an exploratory analysis could be done for regional difference.. A counter to this is the fact that a lot of these predictors are indeed highly correlated - not independent predictors- and I see mention of a princple component analysis but the bulk of the discussion seems to still be looking at each of the CMR predictors individually. Overall, it is hard to discern the real takeaway importance here amongst all the things being compared. Showing associations before disease is diagnosed is a good thing but it is still not clear that disease wasn't present without a diagnosis yet for things like AAA.. The paper seems to be taking on a bit too much as the correlation between CMR indicators and future incidence of cardiac disease in the discussion..

Version 1:

Reviewer comments:

Reviewer #1

(Remarks to the Author)
The authors have specifically responded in appropriate detail to the comments and criticisms of previous reviewers of this paper. The comments and corresponding responses are primarily related to clinical inferences drawn by the authors and statistical questions. In general I think the responses adequately address these points, except for the vague clinical inference highlighted in the Conclusions. “Monitoring both cardiac function and WMH volumes at sub clinical levels, alongside shared risk factor management, may provide valuable insights in the management of heart-brain health,” means little and isn’t justified.
This statement is prominent in the Abstract and Conclusion of the body of the report and hence draws attention that I think detracts from the strengths of the paper. The strength of the study is the extensive and relativley unique collection of cardiac and brain MRI scans, cognitive test results, and rich CVD risk factor and demographic data, along with detailed and appropriate statistical analysis. This empirical information, while primarily ‘descriptive’, is a valuable scientiic contribution and supports publication of this paper. This was not an hypothesis driven paper nor was it intended as a clinical trial of any sort. Hence, I don’t think specific clinical inferences are necessary, though a few might be mentioned in the speculative Discussion of the paper, but not highlighted in the Conclusions. Minor rewording of these sections should suffice.

Reviewer #2

(Remarks to the Author)
I am ok with the author's response. I think it is a bit confusing but ultimately I can see the logic of their approach.

Open Access This Peer Review File is licensed under a Creative Commons Attribution 4.0 International License, which permits use, sharing, adaptation, distribution and reproduction in any medium or format, as long as you give appropriate credit to the original author(s) and the source, provide a link to the Creative Commons license, and indicate if changes were made.
In cases where reviewers are anonymous, credit should be given to 'Anonymous Referee' and the source.

The images or other third party material in this Peer Review File are included in the article's Creative Commons license, unless indicated otherwise in a credit line to the material. If material is not included in the article's Creative Commons license and your intended use is not permitted by statutory regulation or exceeds the permitted use, you will need to obtain permission directly from the copyright holder.
To view a copy of this license, visit https://creativecommons.org/licenses/by/4.0/

Response to Reviewers

We would like to sincerely thank the editor and reviewers for their time and effort in thoroughly reviewing this manuscript. Below are our point-by-point responses to the reviewers' comments. Please note that page numbers referenced in this document correspond to the tracked version of the revised manuscript. Key revisions include improvements to the statistical rationale, which we believe have greatly strengthened the findings and clarified the interpretations in the manuscript.

Reviewer 1 Comments

Comment 1 : The results show expected statistical relationships between cardiac MRI variables, age, and CVD; and brain MRI variables (White Matter Hyperintensities) and cognition. In addition, statistically significant associations were shown between cardiac and brain MRI variables. While the latter results are relatively novel and worth disseminating, their practical significance and inferred clinical utility are quite unclear and weaknesses of the paper.

Response

We appreciate the reviewer's comments in support of disseminating our novel results and have strengthened the explanation of the practical significance and clinical utility of our study. On page 20 we have updated the discussion with additional exposition about the clinical utility.

Updated paragraph

" Our results support the presence of a subclinical relationship between cardiac function and WMH volumes, as well as with executive functioning, supporting the importance of cardiac-health for healthy brain functioning. These associations were stronger in midlife (45- 65 years) compared to later in life (above 65 years), suggesting midlife as a critical age for to improve or maintain heart as well as brain health. While these observations about the importance of midlife health for prolonged healthy living have been made before for lifestyle related factors, we now expand these observations to midlife CMR measurements of cardiac function and structure. Our findings suggest patients identified with subclinical measures of

cardiac function such as lower LVEF that puts them in a borderline range, may be important to monitor in terms of cognitive function and possible later in life adverse brain changes. The localisation of right CMR traits to parietal lobe WMH volumes provides an area of focus for further study to understand the implications on cognition due to the limited amount of research in this area. Our findings are relevant to ongoing considerations about integration of cardiac and cerebral clinical referrals."

Comment 2: First, the effect size, while statistically significant, is relatively small with its practical significance only speculative. This is a not uncommon problem of large population studies with many variables in which some statistically significant results are almost inevitably found, depending on the setting of significance thresholds and multi-test adjustments. "Perhaps to a fault, this reviewer views such results skeptically, wanting additional rationale for any clinical inference".

Response

The manuscript has been updated to better differentiate between small and medium/large associations, and where relevant, to discuss these in terms of potential limitations. We have also updated language on page 16 to clearly distinguish between medium and large associations of CMR and WMH associations. On page 21, we have updated the language to highlight our study identifies subclinical changes that can be used as evidence of the need for early screening. We further describe statistical rationale for clinical inference in response to comment 2 of reviewer 2 (see below).

Updated paragraphs

Page -17

"We observed a consistent and strong association with effect sizes exceeding 5% per standard deviation increase"

"Additionally, we observed associations with effect sizes between 3 and 5% that increased EF associated with lower WMH volumes"

"…our findings show that right heart traits (excluding RVEF) mainly associate with parietal WMH volumes with effect sizes of 2-3%."

Page 22 –

"*Importantly, cognitive associations with increased WMH volumes have relatively small effect sizes in clinical terms. However, these small differences may mark meaningful associations given our sample is disease-free.*"

"This study shows that in people without pre-existing cardiovascular disease, and independently of known CVD risk factors, there is an association between changes in cardiac function and WMH volumes. These findings suggest that monitoring both cardiac function and WMH volumes at sub-clinical levels, alongside shared risk factor management, may provide valuable opportunities for management of heart-brain health."

Comment 3: A step in this supportive direction is often mechanistic, i.e. pathophysiological. The results fit some biological explanatory model. I am not aware of well documented anatomic, physiological, or biochemical mechanisms linking a particular right heard function to white matter integrity in the parietal lobe, though the authors do refer to a vague hypoperfusion hypothesis. While the latter results are relatively novel and worth disseminating, their practical significance and inferred clinical utility are quite unclear and weaknesses of the paper. How might one treat a specific right heart dysfunction that would directly and independently ameliorate parietal white matter deterioration, and cognition. It is particularly difficult to appreciate the clinical significance of such a weak mechanism and its treatment in light of the far larger and general effect of hypertension which still requires attention.

Response

Following the reviewer's suggestion we have provided a more detailed mechanistic insight on the right heart contribution, by addressing key points

1. Explanation of the right heart measurements and cardiac disease linked with cognition
2. Highlighting the previous lack of research considering RV involvement
3. The importance of our novel findings of RV associations with WMH volumes

We have updated paragraph on pages 19-20

<u>Updated paragraph</u>

"The right ventricle has been understudied in heart-brain research as most studies focus on mechanisms arising from the left heart. Our study identifies RVEF, RAV, and RVESV as important RV CMR parameters that associate with parietal WMH burden independent of systemic blood pressure. Increased minimum RAV provides an early and sensitive signal of poor atrial emptying which is relevant given the established link between atrial dysfunction, atrial fibrillation and increased WMH volumes[29 30]. Higher RVESV point towards poor emptying of the ventricle and reduced RV systolic function. In addition to our work, Navarro et al report 'normal' RV internal diameter to be associated with increased WMH volumes using a cohort of 132 individuals [29]. Mean right arterial pressure in a cohort of chronic valvular disease patients also shows associations with increased WMH volumes[30]. Together, these associations indicate early monitoring of right heart function may provide further understanding into the mechanisms of the RV and WMH volumes. Our findings on the specific localisation of these associations to parietal WMH volumes highlights the importance of stratified analysis that previous studies lack. These findings suggest further analysis which could include functional studies and clinical studies focusing on lobar distributions of WMH to better understand these associations."

Reviewer 2 comments

Comment 1: The main area I think could be improved upon is in the statistical analysis. There is a gargantuan amount of stats being run looking at associations but I only see a mention of a division by 10 for correction. Given the equally gargantuan sample size of the study I think that clinically meaningful associations should withstand a more robust multiple comparisons correction considering 29 predictors and 5 outcomes.

Response: We note that the associations between CMR measurements and disease onset is included as a positive control step. Given the high correlation between the considered CMR and brain MRI outcomes (see our response to comment 2) we leveraged principle component analysis (PCA) to identify the appropriate number of multiplicities to account for in a Bonferroni correction. More strenuous multiple testing correction would account for an over correction, in correctly assuming the number of tests are independent of on another[1].

[1] Amand Schmidt, 'Addressing Common Inferential Mistakes When Failing to Reject the Null-Hypothesis', *F1000Research*, 13 (2025), p. 1488, doi:10.12688/f1000research.158434.3.

The methods have been updated to reflect this on page 12

“The primary analysis, the association between CMR traits and cerebral WMH volumes, was corrected for multiple testing applying a Bonferroni correction based on the number of principle components (PCs) needed to explain 90% of the CMR variability; Supplementary Figure 5. Specifically, we found that 10 PCs were needed resulting in a multiple testing corrected p-value of 0.005.”

Furthermore, while we appreciate there is a natural expectation that true association have smaller p-values it is important to note that empirical data represents a mixture of true and false associations. Due to random sampling variability, clinically meaningful associations may never reach statistical significance, while true null associations may show small p-values. As such more conservative multiple testing correction, in the presence of mixtures of true and false associations, does not guarantee a decrease in false positive findings. See for example the illustration[2] (from https://doi.org/10.12688/f1000research.158434.3):

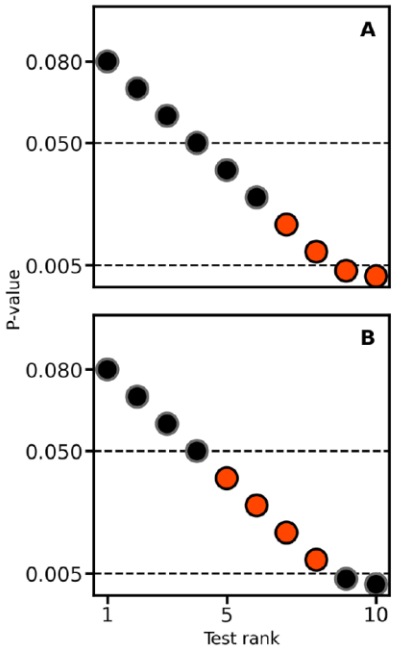

Figure 4. Illustrating the impact of multiple testing correction on the false discovery rate. In each panel, four tests are true positives (orangered) and six are false positives (black). Horizontal lines are drawn at 0.05 and 0.005, the latter reflecting a Bonferroni correction for the 10 applied null-hypothesis tests.

Comment 2: Alternatively, once a positive association is shown for total WMH an exploratory analysis could be done for regional difference

Response

Following the reviewer's suggestion, we report the regional volumes of WMH on page 11 of the manuscript which details volume load of all regions showing highest contributions from the frontal and parietal lobes which can also be found in Supplementary data 5. We, furthermore, note that the regional associations were generally also observed for total WMH volume. This reflects the generally high correlation between total WMH volume and regional volumes (Supplementary Data).

Page 12-13 - *"The median WMH volumes were highest in the frontal lobe (862 mm^3; 432; 1955) with smaller volumes in the subsequent lobes. The parietal lobe (170 mm^3 70; 507) the temporal (223 mm^3 132; 406), occipital (235 mm^3 125; 431) and basal ganglia and thalami regions (171 mm^3 105; 275)"*

In addition, we share the results for all brain lobes associations with cardiac traits in supplementary data 5.

On Page 15 of the discussion, we have highlighted the differences in associations between our findings of the left, aortic and right cardiac traits.

Page 15- " In contrast to left cardiac and aortic traits that have widespread regional WMH associations, our findings show that right heart traits (excluding RVEF) mainly associate with parietal WMH volumes."

Comment 3: A counter to this is the fact that a lot of these predictors are indeed highly correlated - not independent predictors- and I see mention of a principal component analysis, but the bulk of the discussion seems to still be looking at each of the CMR predictors individually

Response

We appreciate the reviewer's suggestion and wish to clarify that the principle components analysis we employed to determine the appropriate level of multiple testing correction required to appropriately account for the mentioned dependencies between traits. To further

clarify this we have now included pairwise correlation estimates between CMR and brain WMH volume measurement, respectively (Supplementary data 4 and 5) and added this to the methods and results. Correlation estimates and rationale have been addressed in comment 2 above.

Page 10-

"Spearman's estimator was employed to determine the correlation between CMR traits (Supplementary Data 5), the correlation between six WMH volumes (Supplementary Data 6), as well as to establish the correlation between non-MRI covariates and CMR traits or WMH volumes (Supplementary Figures 4- 5)."

Page 13-

"Regional WMH volumes excluding basal ganglia and thalami are highly correlated (between 0.72 for parietal and 0.95 for frontal lobe) with total WMH(Supplementary Table S6)."

While valuable as an analytical tool the principle components themselves have limited clinical meaning and as such in the discussion we provide interpretation on the individual traits, of course conscious that these reflect possible wholistic changes in cardiac structure and/or function. The aim was to clearly describe potential mechanistic contributions of each group of measurements to match clinical interpretation.

Comment 4: Overall, it is hard to discern the real takeaway importance here amongst all the things being compared. Showing associations before disease is diagnosed is a good thing but it is still not clear that disease wasn't present without a diagnosis yet for things like AAA.

Response:

We appreciate the reviewer's comments on clarifying disease was not present before diagnosis and to account for that we detailed in the methods that we applied a 30-day window before diagnosis. This is described in the methods on page 8– "*To prevent effects by pre-exiting conditions, we removed participants with a diagnosis of CVD or dementia prior to, or 30 days after, the imaging appointment, specifically we removed: 1,597 individuals with CHD, 198 with HF, 715 with AF, 85 with AAA, and 69 individuals with any dementia".*

This may still not be enough as a 30-day cut off could just seem arbitrary as many of the diseases included have a prodromal stage longer than 30 days. Therefore, in our analysis we

also made sure to remove any outliers in cardiac measurements and have reported mean, median and standard deviation values in supplementary table S5 of the original manuscript. Each measurement, including the lower and upper end of each measurement still falls within clinically ‘normal’ ranges according to reported reference ranges available

We have carried out additional analysis applying a 365-day window which subsequently removes 1,745 individuals with CHD, 230 with HF, 832 with AF, 103 with AAA. This had minimal effect on our originally reported results, which we have included in Supplementary figure 2.

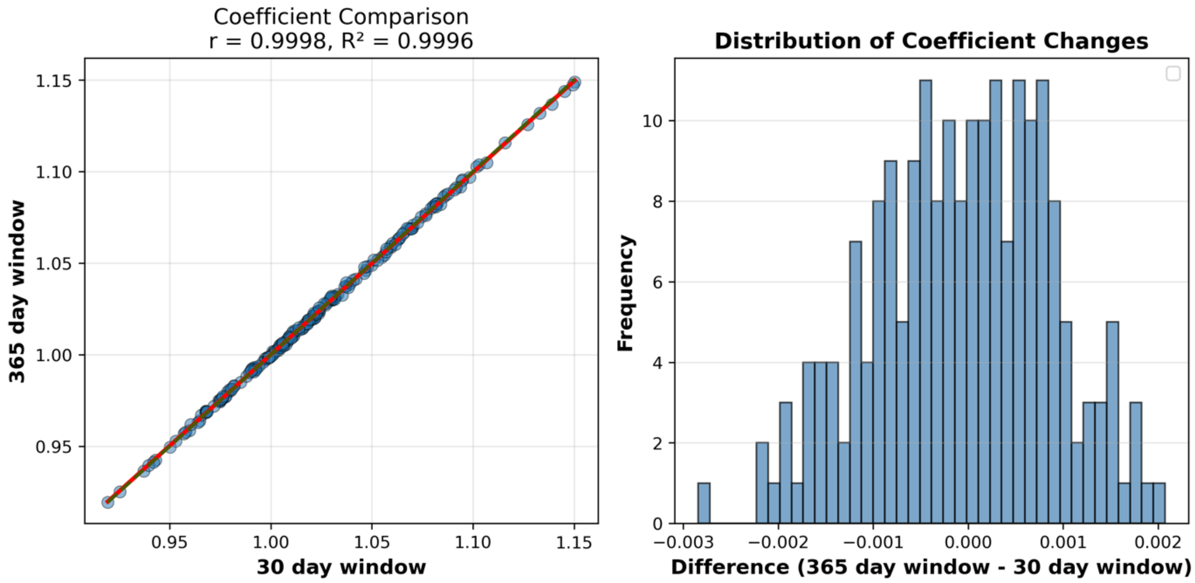

We have subsequently discussed these findings in the results section

Page 8-

“In sensitivity analysis, we further removed participants with a diagnosis 365 days after imaging appointment (Supplementary Figure 2).”

In addition we have included a note in our discussion on page 22

“Second, we report a disease-free study sample based on a 30-day cut off window, free of outliers which may not account for cases where a clear diagnosis had not been made.”

Comment 5: The paper seems to be taking on a bit too much as the correlation between CMR indicators and future incidence of cardiac disease in the discussion

Response

We have proofread over the manuscript and ensured we toned down any inappropriate overinterpretation on correlation and focused our results section on more specific incidence of disease where we had listed multiple.

We have updated the methods on page 11 to show the rationale behind the associations with cardiac disease as empirical validation of the CMR traits. This is now discussed on page 10

"We identified the CMR traits associating with incidence cardiac disease as empirical validation."

We have updated results to associations with incidence disease where the hazard ratio is greater than 1.25 and less than 0.75. We have updated the description on pages 15-16 to remove the phrase "strongly" associated based on these parameters.

Page 15 - "For example, higher minimum and maximum ascending aortic area (associated with AAA)"

Page 16 - " LVM, basal WT, mid WT, apical WT (associated with HF, and AF)

Page 16 - "Similar biventricular associations with WMH volumes were observed for ESV (strongly associated with HF)"

Reviewers' comments:

Reviewer #1 (Remarks to the Author):

The authors have specifically responded in appropriate detail to the comments and criticisms of previous reviewers of this paper. The comments and corresponding responses are primarily related to clinical inferences drawn by the authors and statistical questions. In general, I think the responses adequately address these points, except for the vague clinical inference highlighted in the Conclusions. “Monitoring both cardiac function and WMH volumes at sub clinical levels, alongside shared risk factor management, may provide valuable insights in the management of heart-brain health,” means little and isn’t justified.

This statement is prominent in the Abstract and Conclusion of the body of the report and hence draws attention that I think detracts from the strengths of the paper. The strength of the study is the extensive and relativley unique collection of cardiac and brain MRI scans, cognitive test results, and rich CVD risk factor and demographic data, along with detailed and appropriate statistical analysis. This empirical information, while primarily ‘descriptive’, is a valuable scientiic contribution and supports publication of this paper. This was not an hypothesis driven paper nor was it intended as a clinical trial of any sort. Hence, I don’t think specific clinical inferences are necessary, though a few might be mentioned in the speculative Discussion of the paper, but not highlighted in the Conclusions. Minor rewording of these sections should suffice.

Response:

We would like to thank reviewer 1 for their comments and clearly highlighting the strengths of the study. We have updated the relevant paragraphs:

On page 3, abstract conclusion line 52-54 -*" We show that CMR derived measurements of function and structure are associated with regional WMH volumes implicated in executive functioning and processing speed, independently of known cerebrovascular risk factors, in people without established disease."*

On page 6, introduction line 114-116

"These findings support a relationship between cardiac function and structure, and regional WMH volumes independently of known cerebrovascular risk factors in people without established disease.

"

On page 23, conclusion lines 434-438

"This study utilises an extensive collection of 29 cardiac MRI indices in combination with cognitively relevant regional WMH volume measures and cognitive tests. We demonstrate that in people without pre-existing cardiovascular disease, and independently of known CVD risk factors, there are widespread and regional associations between changes in cardiac and aortic function with WMH volumes."

Reviewer #2 (Remarks to the Author):

I am ok with the author's response. I think it is a bit confusing but ultimately, I can see the logic of their approach.

Response: We thank reviewer 2 for their time and valuable feedback in improving the manuscript
